# Cadaveric Evidence of Complete Transection of the Lumbar Sympathetic Trunk After Extreme Lateral Transpsoas Approach to the Lumbar Spine: A Word of Caution

**DOI:** 10.7759/cureus.14346

**Published:** 2021-04-07

**Authors:** Joe Iwanaga, Tyler Zeoli, Tyler Scullen, Christopher Maulucci, R. Shane Tubbs

**Affiliations:** 1 Department of Neurosurgery, Tulane University School of Medicine, New Orleans, USA; 2 Neurosurgery and Structural & Cellular Biology, Tulane University School of Medicine, New Orleans, USA; 3 Neurosurgery, Ochsner Neuroscience Institute, Ochsner Health System, New Orleans, USA; 4 Department of Anatomical Sciences, St. George's University, St. George's, GRD

**Keywords:** lumber sympathetic trunk injury, surgical training, transpsoas approach, spine surgery

## Abstract

Lateral transpsoas approaches to the lumbar spine are believed to entail less risk of injury to the lumbar sympathetic trunk and plexus than anterior approaches. However, even the lateral approach can occasionally injure the sympathetic trunk. We report a literature review and cadaveric case of complete resection of the left sympathetic trunk at L3 following lateral transpsoas approach performed by a well-trained spine surgeon. A left lateral approach to the lumbar spine for a two-level total discectomy at L3-L4 and L4-L5 was undertaken on a fresh-frozen cadaver by an experienced spinal surgeon. The procedure followed standard spinal technique under fluoroscopy guidance. The cadaver was placed in a right lateral position and an operative corridor to the lateral aspect of the psoas major muscle was developed. Blunt dissection was carried through the muscle and standard total discectomy was completed at the target levels. Following the procedure, the lumbar spine and adjacent structures were anatomically dissected. It was found that the sympathetic trunk had been completely transected at the L3 level during the surgical procedure. Other major structures such as the femoral nerve, obturator nerve, and roots of the lumbar spinal nerves had not been injured. The above case highlights the proximity of the sympathetic trunk to lateral transpsoas approaches and the possibility of injury to it. We review the literature on postoperative cases of lumbar sympathetic dysfunction (SD) following such procedures and posit that some of these are due to direct iatrogenic injury.

## Introduction

Extreme and direct lateral transpsoas approaches have grown in popularity as minimally invasive corridors for managing mid and upper lumbar spinal pathologies [1]. Ideally, the approach avoids peritoneal and retroperitoneal structures by dissecting through the muscles of the abdominal wall (internal and external obliques, transversus abdominis) and the lateral axial skeleton [1]. Deviation of the dissection plane into the retroperitoneum or peritoneum entails a risk of injury to urinary and gastrointestinal tract structures, and targeting of the more caudal vertebrae and disc spaces entails a greater risk of injury to the lumbar and lumbosacral plexuses if not accounted for during pre-operative planning [2-4]. Therefore, anatomical studies are continuing to seek ways of minimizing the risk of injury [5-8]. As with any surgery, complex minimally invasive spinal surgery necessitates strong anatomical knowledge in addition to technical capability [1]. Nevertheless, even the skilled and well-trained surgeon must be prepared for the unexpected owing to anatomical variations or unusual circumstances. We report a case of complete transection of the lumbar sympathetic trunk demonstrated by anatomical dissection following a lateral transpsoas surgical approach to the mid lumbar spine using a cadaveric specimen.

## Case presentation

As part of a surgical demonstration course, a left lateral transpsoas approach to the mid lumbar spine for a two-level total discectomy at L3-L4 and L4-L5 was undertaken on a fresh-frozen Caucasian male adult cadaver (middle-sized) by an experienced spinal surgeon (over 10 years in practice) who was a participant in the course. The procedure followed normal operative spinal technique under fluoroscopy guidance. The cadaver was placed in the right lateral position with standard adjustments to open the lateral lumbar corridor. The skin was incised per standard procedure and dissection continued down through the lateral abdominal wall to the lateral aspect of the psoas major muscle. Following fluoroscopic confirmation of position, blunt dissection was carried through the muscle and successive dilators, and self-retaining retractors were subsequently placed to visualize the target disc spaces. The L3-L4 and L4-L5 disc spaces were then subjected to annulotomy and total discectomy.

**Figure 1:**
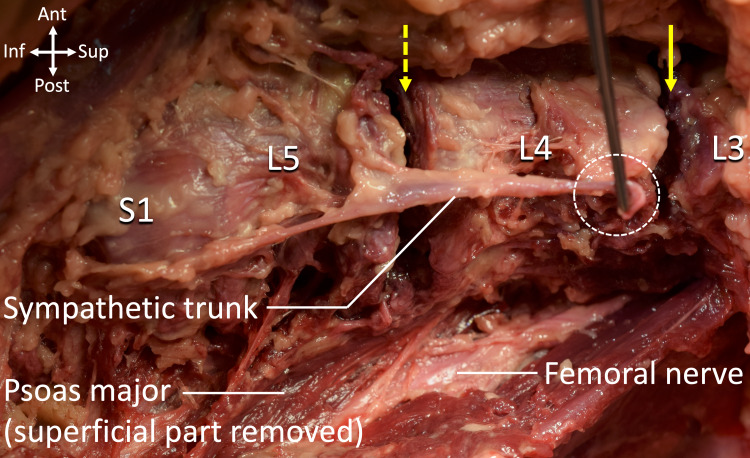
Cadaveric specimen following direct lateral transpsoas approach for total discectomy of the L3-L4 (arrow) and L4-L5 (dashed arrow) intervertebral discs. Note: The lumbar sympathetic trunk was completely transected at the L3 level.

Following the procedure, the lumbar spine and adjacent structures were anatomically dissected via a retroperitoneal anterior approach. It was found that the sympathetic trunk had been completely transected at the L3 level during the surgical procedure (Figure 1). Other major structures such as the femoral nerve, obturator nerve, and roots of the lumbar spinal nerves had not been injured.

## Discussion

The lumbar sympathetic trunk is connected to the ventral ramus of the lumbar spinal nerves via the gray and white rami communicantes and usually contains four interconnected ganglia sitting in the extraperitoneal connective tissue on the anterolateral surface of the lumbar vertebrae along the medial border of the psoas muscle found the disc spaces at L2-L3 and L3-L4 to be the levels at which the lumbar sympathetic ganglia were usually located, an area where anatomical variations are frequent [8-12].

Although injury to the sympathetic trunk is not fatal, it can carry significant morbidity secondary to deficits in a patient's quality of life [11,12]. The main advantage of the lateral transpsoas approach over the anterior transperitoneal, retroperitoneal, or prepsoas approaches is that there is no need to expose the great vessels, abdominal viscera, or sympathetic trunk [13]. The lateral approach also retains the benefits of minimally invasive surgery, requiring a smaller incision, less abdominal wall injury, and shorter hospital stays [13]. However, one major risk of the lateral approach, particularly in the caudal lumbar spine, is an injury to the spinal nerves as they exit via the neural foramina and course through the psoas muscle to form the lumbar plexus [14]. 

Lumbar sympathetic dysfunction (SD), carrying multiple potential clinical presentations such as dislocation, dysesthesia, reduced perspiration, increase in skin temperature, and swelling of the lower limb, has been reported in 4% of patients undergoing lateral transpsoas approaches at T12-L5, though this is 15% lower than the rate of injury documented during anterior approaches at the same levels [15]. Additional possible complications include retrograde ejaculation due to injury to the sympathetic plexus (well known in the urology literature), which occurs more often during dissection of the anterior longitudinal ligament in anterior approaches [16].

As mentioned, the lumbar sympathetic trunk usually runs along the medial border of the psoas [9,10]. This is one reason why many lateral approaches use the transpsoas corridor [1-4]. In the lower lumbar spine, anterior dissection can also injure the superior hypogastric plexus [17]. Clinically, the etiology of perioperative neuropathy can be difficult to confirm, as it is not always feasible to determine what occasioned the nerve injury (compression, traction, partial or total resection, etc.) [17]. Although we recognize that cadaveric and living tissues are very different in consistency, appearance, and physical reaction to surgical manipulation and dissection, we consider it plausible that perioperative lumbar SD has been underreported in patients undergoing minimally invasive lateral spinal procedures. Either anatomical variation of the course of the sympathetic trunk or technical issue might have caused the injury in the present case. This might be a good example that even well-experienced surgeons need to revisit the anatomy and basic technique so that iatrogenic injuries might be avoided.

## Conclusions

We described a cadaveric report of iatrogenic lumbar sympathectomy following lateral exposure of the mid-lumbar spine for two-level discectomy by an experienced spine surgeon. To our knowledge, this is the first report of a cadaveric case demonstrating complete resection of the lumbar sympathetic trunk after a lateral transpsoas approach to the lumbar spine. This complication and its perioperative clinical consequences may be difficult to diagnose and avoid, even in the hands of experienced spinal surgeons. We urge spinal surgeons to further consider the lumbar sympathetic trunk and its anatomy in perioperative planning for lateral approaches.
